# A dataset of tissue-specific gene expression dynamics during seed development in *Brassica*

**DOI:** 10.1038/s41597-025-05082-w

**Published:** 2025-05-16

**Authors:** Hugh Woolfenden, Laura Siles, Martin Vickers, Burkhard Steuernagel, Richard J. Morris, Rachel Wells, Smita Kurup

**Affiliations:** 1 Department of Computational and Systems Biology, John Innes Centre, Norwich Research Park, Norwich, NR4 7UH UK; 2 Plant Sciences and the Bioeconomy, Rothamsted Research, Harpenden, AL5 2JQ UK; 3 Department of Crop Genetics, John Innes Centre, Norwich Research Park, Norwich, NR4 7UH UK

**Keywords:** Plant sciences, Plant embryogenesis

## Abstract

In oilseed crops, e.g. oilseed rape (OSR; *Brassica napus*), a key developmental process is seed maturation, during which the embryo transitions from the early, globular state to the mature state. Seed development involves cell division, differentiation, and oil accumulation in specific tissue types (embryo, endosperm, and seed coat). These developmental processes impact seed quality and oil yield. High quality RNA from *Brassica* spp. seed tissues, from heart to mature developmental stages, was obtained using previously reported methods for five Brassica genotypes comprising winter, semi-winter and spring OSR varieties, a *B. napus* heritage kale and a rapid-cycling double-haploid *Brassica oleracea* line. RNA-seq was performed on 240 sets of samples. The resulting dataset contains detailed spatio-temporal expression profiles during seed development. In addition to the repository data, we provide easy access to this through the Seed Oilseed Rape Developmental Expression Resource (SeedORDER), which enables users to search for genes of interest and visualise expression patterns. Knowledge of where and when genes are expressed during seed development will inform future breeding efforts.

## Background & Summary

The eudicot family, Brassicaceae, comprises the economically important *Brassica* genus, which includes vegetables and oil crops, such as *Brassica napus* (e.g. oilseed rape (OSR)), *Brassica oleracea* (e.g. cabbage, cauliflower, broccoli) and *Brassica rapa* (e.g. pak choi and turnip^1,2^). *Brassica napus* has multiple crop types including winter, semi-winter and spring OSR, root vegetables, kales and animal fodders, which are mainly grown for animal feed, vegetable oil and biodiesel production^1,3^. Different environmental requirements are distinguished in these crop types that influence in which geographical region they are cultivated. Winter OSR cultivars require prolonged cold periods to promote the onset of flowering in a process known as vernalisation; they are sown in the autumn and flower in spring. Semi-winter OSR, respond to but do not require cooler temperatures and therefore are cultivated in geographical regions with moderate winter temperatures^1,4^. Spring OSR cultivars have a shorter life cycle compared to winter OSR varieties and are sown at the end of winter as vernalisation is not required. The diploid *B. oleracea* comprises vegetable crops that are mainly grown for their leaves, stems and flowers^5^. Like *B. napus*, some *B. oleracea* crops require vernalisation for flowering while others are non-vernalisation dependent^6^.

Derived from the same ancestral species as Arabidopsis, *B. napus* (AACC genomes) was formed by the hybridisation between *B. rapa* (AA genome) and *B. oleracea* (CC genome) about 7500 years ago^7^. The merger of these two progenitor genomes along with duplication and triplication events that *Brassica* spp. ancestors experienced, has led to a large number of duplicated regions in the *B. napus* genome^8^. Consequently, Arabidopsis genes can have multiple orthologs in *Brassica* spp., some of which may have functionally diverged during evolution and domestication. Sub- and neo-functionalisation can hamper efforts to transfer the rich knowledge of Arabidopsis to Brassica crops. Studying gene expression dynamics across *B. napus* development can help identify which orthologs are important for different processes.

We conducted an RNA-seq time-series study in five different *Brassica* spp. genotypes during seed development. The genotypes were chosen to represent different environmental requirements and included a winter OSR, semi-winter OSR, spring OSR a heritage rape kale (*B. napus*), and the rapid-cycling double-haploid line, AGDH1012 (DH1012; *B. oleracea*). A total of 16 different tissue-types were collected^9^, thereby generating a comprehensive reproductive dataset which also includes earlier vegetative stages to provide baseline information. For seed development, the stages of cell division, cell differentiation and oil accumulation as well as maturation were included in the study, representing seed development from early stages to maturity. The embryo, endosperm and seed coat were analysed as independent tissue types to capture the distinct mRNA profiles at different stages of development^10–15^.

## Methods

### Plant material

Samples were collected from the *B. napus* genotypes, Express 617 (a modern winter cultivar), Zhongshuang 11 (ZS11; a semi-winter cultivar), Ragged Jack (a heritage kale cultivar), Stellar (a spring cultivar), together with samples from the *B. oleracea* line, AGDH1012 (DH1012; rapid-cycling, double-haploid line from a cross between *B. oleracea alboglabra* ‘A12’ and *B. oleracea italica* ‘Green Duke’).

### Plant growth

Seeds were germinated and the plants grown under controlled environmental conditions with a 16 h photoperiod at 18 °C/15 °C day/night temperatures, respectively, under 400 W HQI lighting (225 µmol m^−2^s^−1^). The relative humidity was maintained at 70% day and night. At 3 weeks old, the plants from the winter and semi-winter genotypes were transferred to rooms at colder temperatures that induce the process of vernalisation. After three weeks (ZS11), six weeks (Express 617) or 12 weeks (Ragged Jack) of cold treatment, plants were returned to the controlled environment rooms until maturity.

### Sample preparation

For each *Brassica* spp., tissue collections, manual pollinations and dissections were performed as previously described^9^ to obtain embryo, endosperm and seed coat seed-specific tissues, together with leaves and floral apex (at 21 days after sowing), ovules and ovary walls. As speed of development varies between accessions, samples were carefully staged for all accessions. Buds were collected 24 h before anthesis to obtain ‘pre-fertilization stage’ ovules and ovary wall tissue. Flowers were manually pollinated and tagged to determine the days after pollination. Embryos within the seed were then visually checked to ensure they were at the correct stage of development prior to sampling and extraction. The embryo, endosperm and seed coat tissues were collected at heart, torpedo and green stage, with embryo and seed coat also collected at mature stage. Silique valve walls at “embryo green stage” were also collected. Three biological replicates were performed for each time point and tissue type. At each time point, relevant tissue was extracted using sterile forceps over a clean, sterile tile placed on a bed of ice within a 14 cm diameter plastic petri dish to ensure the samples were cold throughout the dissection procedure. The samples were immediately frozen in liquid nitrogen and stored at −80 °C. A list of the tissue types and development stages is given in Table 1.

**Table 1:** Sampled tissues together with their development stage.

Tissue	Dev. Stage
Apex	Day 21
Leaf	Day 21
Gynoecia wall	Pre-fertilisation (24 h before anthesis)
Ovules	Pre-fertilisation (24 h before anthesis)
Embryo	Heart stage
Seed coat	Heart stage
Endosperm	Heart stage
Embryo	Torpedo stage
Seed coat	Torpedo stage
Endosperm	Torpedo stage
Embryo	Green stage
Seed coat	Green stage
Endosperm	Green stage
Silique wall	Green stage
Embryo	Mature stage (not fully dry)
Seed coat	Mature stage (not fully dry)

Eight distinct tissues were sampled across development, resulting in 16 different tissue/development stage combinations.

### RNA extraction details

High quality total RNA was extracted as previously described^9^. All the collected valve tissue for each replicate was pooled together and finely powdered by grinding in liquid nitrogen in an autoclaved mortar and pestle. TRIzol reagent (ThermoFisher Scientific, Waltham, USA) was used for the extraction of valve tissue RNA as previously described^9^ for green stage seed coat. The RNA was subsequently treated for DNAse using the TURBO DNA-free kit (ThermoFisher Scientific, Waltham, USA). RNA integrity number (RIN) was determined using an Agilent 2100 Bioanalyzer (Agilent Technologies, Inc.) at Rothamsted Research. Total RNA samples were processed by Novogene (Beijing, China). Libraries were sequenced on the Illumina NovaSeq 6000, resulting in 150 base pair paired end reads.

### Gene expression quantification

We processed a total of 240 RNA-seq 150 bp paired-end read samples, comprising five genotypes (four *B. napus* and one *B. oleracea*) and 16 tissues (see Table 1 for details). All raw RNA-seq sequence data have been deposited in the European Nucleotide Archive (ENA) (see Data Records). We performed quality control for all samples using FastQC (version 0.11.9) before analysing the samples using an RNA-seq alignment pipeline consisting of Trimmomatic (version 0.39)^16^, HISAT2 (version 2.1.0)^17^ (with additional options “–pen-noncansplice 20–mp 1,0”^18^) and StringTie (version 2.1.1)^19^. Trimmomatic trims adapter sequences and quality checks RNA-seq sequencing reads, HISAT2 is a splice-aware aligner, which is able to align sequencing reads that span a splice site to genomic reference sequences, with samtools (version 1.17)^20^ used to filter the results and extract the uniquely mapped reads. StringTie is then used to quantify the gene expression from the aligned sequencing reads. The *B. napus* and *B. oleracea* genotypes were aligned to Darmor-bzh (v4.1)^7^ and TO-1000^21^, respectively. The alignment script is available in FigShare^22^ (see Code Availability). The mean number of raw reads across all genotypes and tissues was 29.5 M reads, with the minimum and maximum numbers, 24.4 and 42.2 M reads, respectively. The mean alignment rate was 88.3% with the mean alignment rate for uniquely mapped reads equal to 73.0%. The alignment statistics for all samples is available in FigShare^22^ (see Data Records).

### Gene homology and aliases

We obtained the Arabidopsis aliases from the cDNA FASTA file for Araport 11^23^. We downloaded the homology data from Ensembl Plants Compara 112^24^ and extracted the Arabidopsis to *B. napus*, and the Arabidopsis to *B. oleracea* mappings. From this data, we also retained the *B. napus* and *B. oleracea* paralogs if the Brassica gene was orthologous to an Arabidopsis gene or where the average of the query and target sequence identities was 65% or more. The homology and gene aliases are tabulated in FigShare^22^.

## Data Records

The raw RNA-seq data (FASTQ files) in this study have been deposited in the European Nucleotide Archive (ENA) under accession number PRJEB86494^25^. Tabulated sample metadata are given in Supplementary Table 1 and are also provided with the data at the ENA^25^. The expression data for each genotype is also available in FigShare^22^ together with a report of the alignment statistics. In addition to the repository data, expression data can be accessed, downloaded and visualised in our seed expression browser, SeedORDER^26^.

## Technical Validation

### Quality control assessment of sample transcript expression by heatmap and principal component analysis

**Fig. 1:**
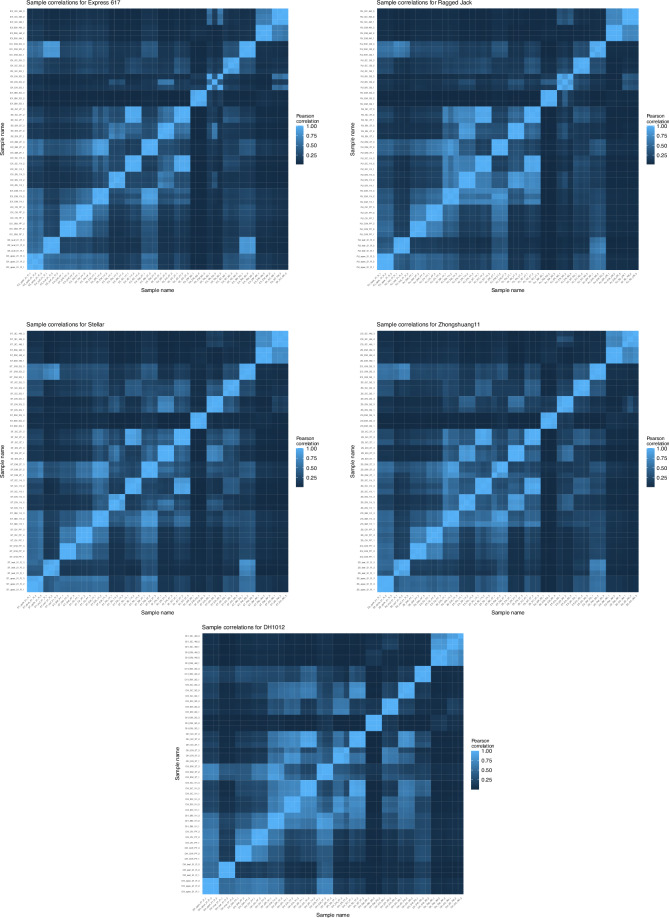
Genotype-specific correlation analysis. Each heatmap shows the Pearson correlation coefficient between the samples for each accession. The sample names encode the accession, tissue, timepoint/stage and replicate, e.g., DH_SC_4M_3 is DH1012 Seed Coat at Mature stage (replicate 3). Full details are given in Supplementary Table 1.

**Fig. 2:**
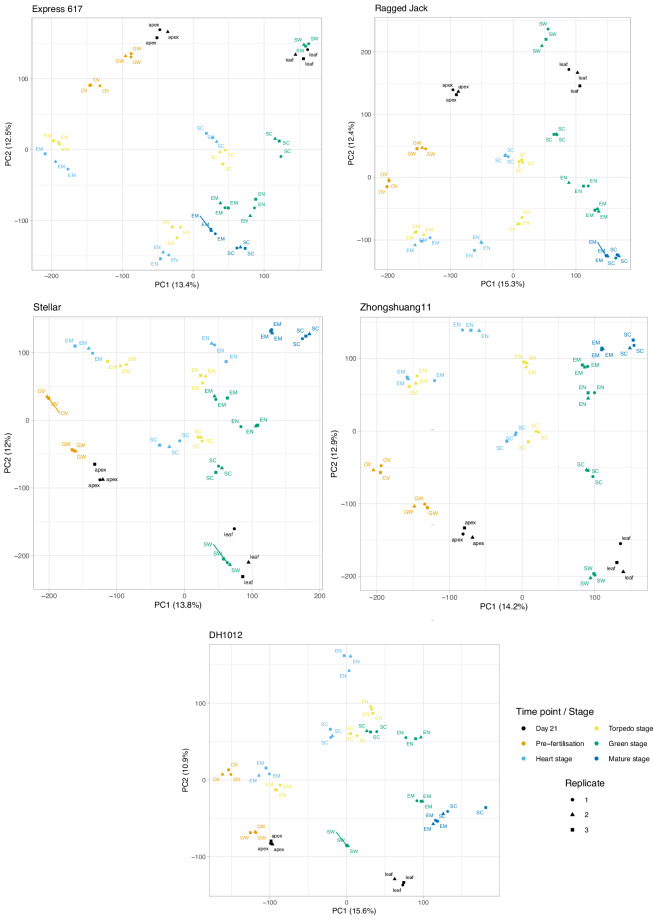
Genotype-specific principal component analysis. The first two principal components are shown for each accession with the explained variance given in parentheses. The stages are colour-coded to aid visualisation with the figure legend shown at the bottom-right. EM – embryo, EN – endosperm, GW – gynoecia wall, OV – ovules, SC – seed coat, SW – silique wall.

Firstly, we examined the intra- and inter-sample correlations to assess the replicate quality and tissue specificity (Fig. 1). For each accession and tissue the three replicates show high correlation demonstrating the quality of the replicates. To further examine the sample expression and visualise the development time-course, we performed principal component analysis and found the three replicates clustered together for all tissues in each of the genotypes, again highlighting the high integrity of the replicates (Fig. 2). We then examined the tissues and found that each of the pre-fertilisation tissue clusters (apex, leaf, gynoecia wall and ovules) were separate to the seed-specific samples (embryo, endosperm and seed coat). In addition, the silique walls (harvested at green stage) also usually clustered closest to the leaf samples. For seed tissue, the embryo and endosperm at heart and torpedo stage clustered closely compared to green stage tissue types in all the genotypes. The seed coat exhibited the same trend apart from in the genotypes Stellar and DH1012 where the clusters for the stages were closer together. Overall, this indicated that the heart and torpedo stages are more similar to each other than to green stage, and that there is a specific transcriptomic transition towards green stage. These results were as expected as in heart and torpedo timepoints share similar regulatory pathways compared to green stage. The mature embryo and the mature seed coat clusters were close in all genotypes possibly highlighting the convergence of gene expression profiles in those tissues. These results indicate the specialised nature of their transcriptomes and the progression of seed maturation in all the tissue types. Further principal component plots are available on FigShare^22^ together with a breakdown of the variance explained by each component.

In summary, the heatmap and principal component analysis demonstrated that all biological replicates were in close proximity. The developmental trajectory is given by the sequence of tissue-specific clusters, suggesting that seed dissections allowed us to detect tissue-specific gene expression profiles.

### Expression from known reference genes for seed developmental tissues

**Fig. 3:**
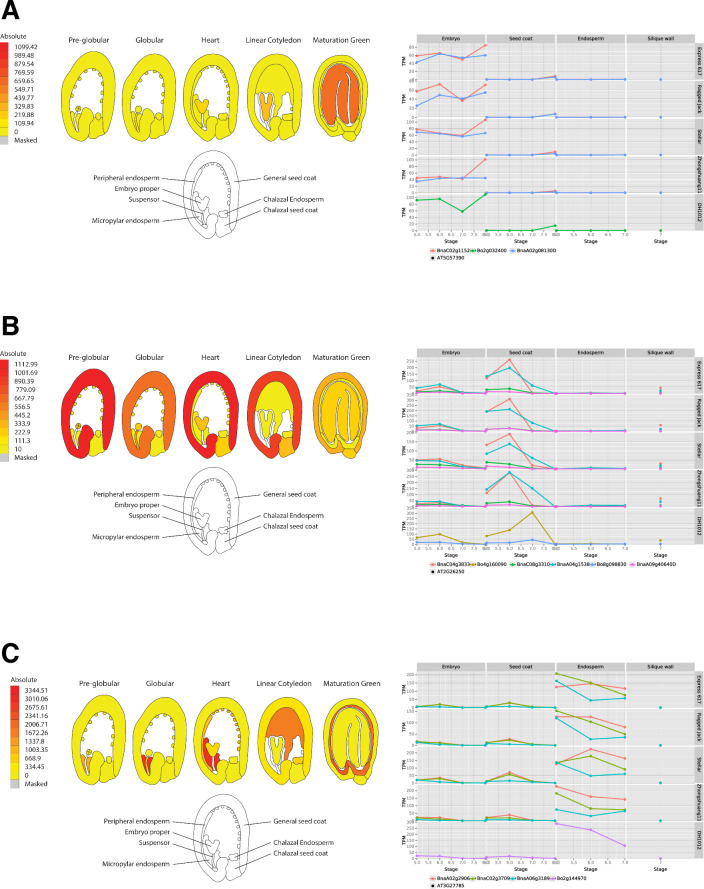
Tissue-specific expression comparisons. Comparisons between genes in the Arabidopsis expression browser^23,27–29^ and the orthologs of each gene in SeedORDER for (**A**) *AIL5* (AT5G57390) which is embryo specific, (**B**) *FDH* (AT2G26250) which is seed-coat specific, and (**C**) *MYB118* (AT3G27785) which is specific to the endosperm. The expression time-courses shown on the right are from SeedORDER^26^ with heart, torpedo, green and mature stages corresponding to the numbers 5, 6, 7 and 8, respectively.

To corroborate the gene expression profiles within our transcriptomes with known seed-specific tissue expression we compared expression data with the Arabidopsis expression browser^23,27–29^ for known seed marker genes. Corresponding tissue expression between the model and the crop confirm the expected tissue specific expression pattern is retained in our dataset demonstrating the tissue specific nature of our RNA-seq dataset. Within Arabidopsis, *AINTEGUMENTA-LIKE 5* (*AIL5*, AT5G57390), is known to be expressed in the seed embryo across development. Two homologues of *BnaAIL5*; *BnaA02g08130D* and *BnaC02g11520D* (*B. oleracea* orthologue *Bo2g032400*) are annotated within the Brassica reference genomes. Expression of the Brassica *AIL5* orthologs was consistent with the known expression of *AIL5* in Arabidopsis (Fig. 3A). Notably, expression within the embryo rose towards maturity while expression in seed coat and endosperm remained very low, thus confirming the tissue specificity of our embryo samples. *FIDDLEHEAD* (*FDH*, AT2G26250) is expressed within the Arabidopsis seed coat and has four homologues in *B. napus*, BnaA04g15380D, BnaA09g40640D, BnaC04g38330D and BnaC08g33100D (*B. oleracea* orthologues Bo4g160090 and Bo8g098830). Here we found high expression of the A04 and C04 homologues, particularly at the heart and torpedo stages of seed development, which is consistent with *FDH* expression in Arabidopsis (Fig. 3B). Peak expression of the C04 homologue occurred later, at the green seed stage, in *B. oleracea*. Furthermore, low expression of *FDH* was observed in the embryo. Finally, we checked the expression of the endosperm-specific gene of myb transcription factor *MYB118* (AT3G27785) which is known in Arabidopsis to have high expression only in the endosperm throughout development. Four homologues are present within the *B. napus* reference, BnaA02g29060D, BnaA06g31890D, BnaC02g37090D (*B. oleracea* orthologue Bo2g144970) and unanchored BnaCnng73990D. This unanchored homologue was given a lower confidence score for homology due to the reduced identity and coverage levels, and expression was not detected in any tissue. This suggests BnaCnng73990D is a pseudogene. A second C genome copy was not annotated in *B. oleracea*. Again, the expression profiles of the other homologues were consistent with the Arabidopsis gene (Fig. 3C).

## Supplementary information

Supplemental Table 1

## Data Availability

The alignment script together with the software and versions used throughout, and as described in Methods, are provided in FigShare^22^.
